# Synthetic Biology and Biomaterials Strategies to Deceive the Tumor Microenvironment in CAR‐T Immunotherapy

**DOI:** 10.1002/smll.202506429

**Published:** 2025-09-01

**Authors:** Grazia Marsico, Sudarshan GC, Velia Siciliano

**Affiliations:** ^1^ Synthetic and Systems Biology Lab for Biomedicine Istituto Italiano di Tecnologia‐IIT Largo Barsanti e Matteucci Naples 80125 Italy

**Keywords:** biomaterials, CAR‐T, delivery systems, ex vivo mechanical models, immunotherapy, solid tumor microenvironment, synthetic biology

## Abstract

Chimeric antigen receptor (CAR)‐T cell immunotherapy has emerged as a groundbreaking approach in cancer treatment, offering new hope across various malignancies. However, its success against solid tumors remains limited due to critical challenges, including off‐tumor, on‐target toxicity, immune resistance, poor T cell infiltration into the tumor microenvironment (TME), and T cell exhaustion. In response, interdisciplinary innovations in synthetic biology and biomaterials are redefining how can be engineer smarter, more responsive CAR‐T cells. Recent advances have introduced biomaterials not only as precision delivery vehicles but also as artificial antigen‐presenting cells (APCs), high‐throughput screening platforms, and tools to replicate the complex biomechanical landscape of the TME. This review highlights these cutting‐edge strategies, emphasizing how biomaterials and synthetic circuits can aid studies and strategies to enhance CAR‐T cell efficacy in solid tumors while minimizing adverse effects. Future directions at the intersection of tumor‐inspired biomaterials and T cell engineering, envisioning a new generation of CAR‐T therapies tailored to overcome the formidable barriers of the TME are also explored.

## Graphical abstract

A. Synthetic biology and biomaterials have emerged as tools to study and facilitate the treatment of solid tumors wi‐th chimeric antigen receptor‐CAR‐T cells. These strategies aim to resist the tumor microenvironment‐TME that presents cellular, biochemical, and mechanical immunosuppressive signals that hinder CAR‐T efficacy. B. The field of biomaterials has contributed to the development of hydrogels, nanoparticles (NP)s, or the combination to improve CAR‐T trafficking and proliferation directly injected into the tumor site. C. NPs have also been engineered as antigen presenting cells (APCs) to improve antigen presentation in the context of tumor antigen heterogeneity. D. 2D, 3D gel systems as well as patient tumor derived organoids (TDOs) and tumors on a chip, are promising tools that help to gain mechanistic insight of CAR‐T cell response to the TME stimuli, including the mechanical forces that remain understudied.

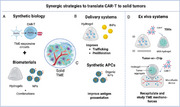

## 1 Introduction

**Figure 1:**
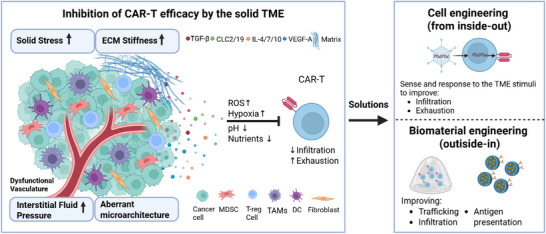
TME features that inhibit CAR‐T and potential solutions offered by cell and biomaterial engineering. The solid TME presents cellular, biochemical, and physical/mechanical compartments. Besides cancer cells, the TME presents myeloid‐derived suppressor cells (MDSCs), T‐regulatory cells (T‐reg), tumor associated macrophages (TAMs), dendritic cells (DCs), and fibroblasts. These cells secrete immune suppressive cytokines and chemokines such as TGF‐β, CLC2, CLC19, IL‐4, IL‐7, VEGF‐A. Other biochemical features are oxidative stress (ROS increase), hypoxia, acid pH, and the lack of nutrients. The physical environment is characterized by dysfunctional vasculature, increased ECM deposition, and aberrant microarchitecture. All these elements contribute to creating mechanical forces such as solid stress, matrix stiffness, and interstitial fluid pressure. The TME elements act in concert in inhibiting CAR‐T cell efficiency, by preventing infiltration and inducing exhaustion. Strategies to solve this challenge involve engineering CAR‐T with synthetic receptors and/or circuits to improve infiltration and revert exhaustion. Other strategies consist of engineering biomaterials as delivery systems to improve infiltration and trafficking and as artificial presenting cells that improve antigen presentation to CAR‐T. Figure created with biorender.com.

Cancer is one of the most lethal diseases with 20 million new cases and 9.7 million deaths worldwide only in 2022. The frontline therapies such as the combination of surgery, radiotherapy and chemotherapy, still present several challenges including tumor relapse, drug resistance, and off‐target toxicity. T cell‐based immunotherapy represents a revolutionary breakthrough in cancer treatment as it elicits a specific anti‐tumor immune response, facilitating its elimination.^[^ ^1^ ^]^ In the past decade, different classes of T cells have been tested for cancer therapy, such as T cell receptor (TCR) T cells, tumor infiltrating lymphocytes (TILs), and chimeric antigen receptor (CAR)‐T cells. CAR‐T patient‐autologous cells are engineered to express a chimeric receptor that guides lymphocytes to bind and eliminate cancer cells bearing a target ligand, usually a specific overexpressed membrane protein.^[^ ^2^, ^3^ ^]^ Notably, CAR‐T cells have shown striking clinical efficacy for many blood malignancies, to the point that CD19‐targeted CAR T cells have been the first genetically engineered therapeutic approved by the Food and Drug Administration (FDA) in 2017.^[^ ^4^, ^5^ ^]^ Since then, another ten CAR‐T products have been launched on the market to treat acute lymphoblastic leukemia (B‐ALL), diffuse Large B‐cell lymphoma (DLBCL), mantle cell lymphoma (MCL), multiple myeloma (MM), follicular lymphoma (FL)^[^Among them seven products are approved by US FDA, two by the Chinese National Medical Products Administration (NMPA), and two by the Indian Central Drugs Standard Control Organization (CDSCO).^[^ ^6^ ^]^ Furthermore, over 170 clinical trials are currently ongoing (ClinicalTrials.gov), indicating the wide interest of the scientific community in investigating further applications of this promising tool. Yet, there are key issues associated with CAR‐T therapy including potential severe toxicity, tumor antigen escape, limited infiltration within the tumor, and T cell exhaustion which leads to the loss of the effector cytotoxic function.^[^ ^7^, ^8^ ^]^ The origin of these phenomena maybe lies in the intricate net of signals imparted by the tumor microenvironment (TME),^[^ ^9^ ^]^ a complex immunosuppressive niche composed of dysregulated cytokine levels, stiffened extracellular matrix (ECM) which raises physical barriers and specific mechanical forces, aberrant blood vessels, immune and stromal cells contributing to maintain a hostile scenario for CAR‐T cells (**Figure** **1**).^[^ ^10^ ^]^ Interdisciplinary approaches like synthetic biology,^[^ ^11^ ^]^ and technologies based on biomaterials^[^ ^12^ ^]^ and microfluidic devices can facilitate the studies for improved cell therapies able to address these issues (Figure 1).^[^ ^13^ ^]^ Synthetic biology engineers smarter CAR‐T cells via the integration of a genetic circuit able to translate inputs into specific cell response that can increase their life span and infiltration, counteract exhaustion, and increase the tumor killing efficacy.^[^ ^14^, ^15^, ^16^, ^17^ ^]^ While synthetic biology acts “from inside” of the cells (Figure 1), biomaterials aim at augmenting the CAR‐T efficacy “from outside” by providing a TME‐based support. The application of biomaterials includes hydrogel or nanoparticle employed as CAR‐T in vivo delivery systems,^[^ ^12^, ^18^, ^19^ ^]^ in vitro 3D systems to activate and expand CAR‐T prior administration in patients, combination of biomaterials and microfluidic devices to gain insight into the impact of the TME mechanical forces on CAR‐T cell functions.^[^ ^12^, ^20^, ^21^ ^]^ This review provides an overview of the most recent bioengineering strategies implemented to produce a new class of CAR‐T that can tear down the TME obstacles. First, we focus on the synthetic biology strategies to engineer “from the inside” CAR‐T cells. Second, we provide an insight on how biomaterials and tissue engineering can give an “outer” help to the CAR‐T cause. In perspective, the synergistic cooperation of these two fields can further revolutionize immune‐cell therapy and achieve outstanding advancements in cancer treatment.

## 2 Engineering cells “From Inside‐Out”: Synthetic Biology to Improve CAR‐T Efficacy

**Figure 2:**
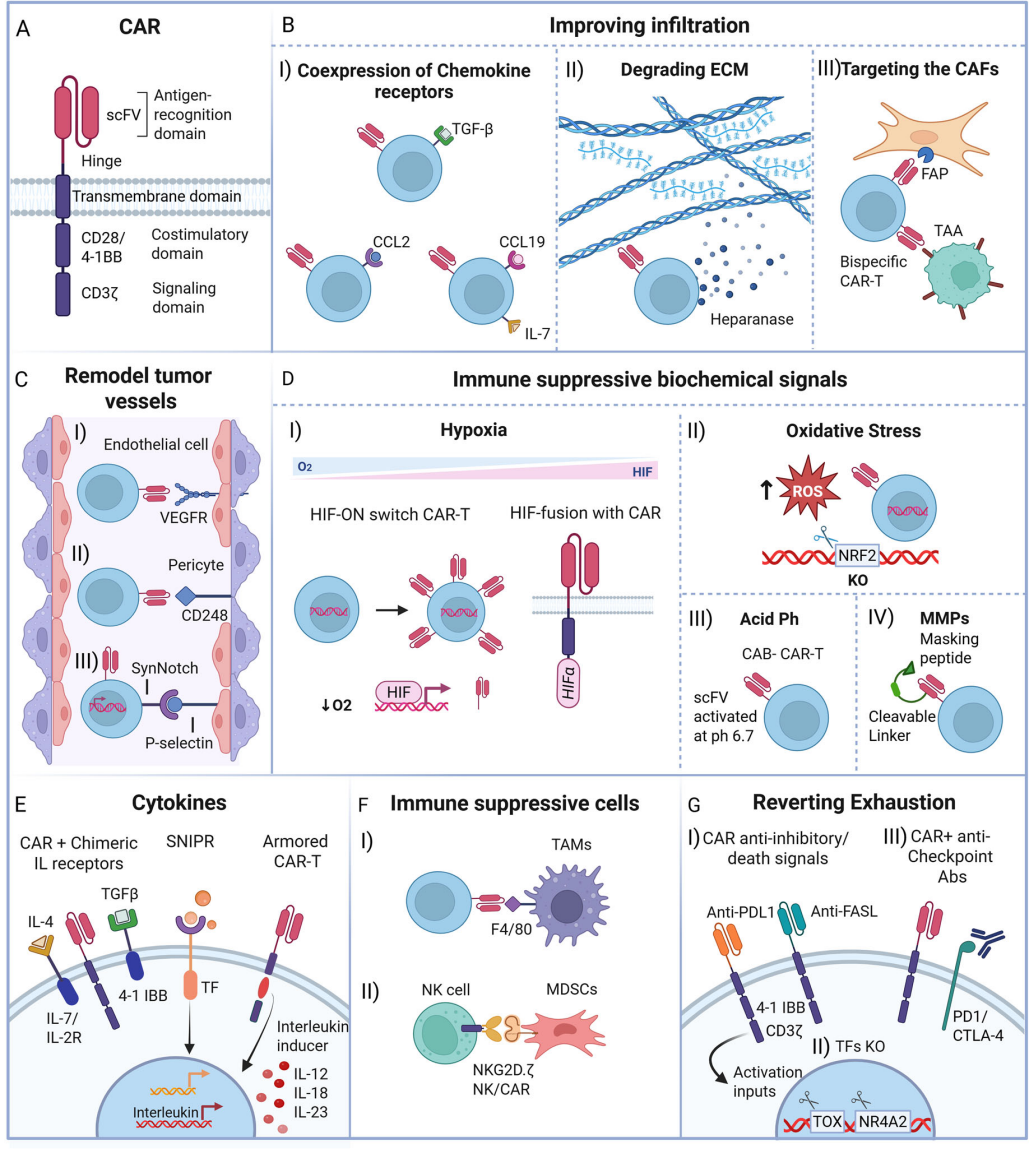
Overview of CAR‐T structure and synthetic biology strategies to engineer TME responsive CAR‐T. A) Most CARs consist in an extracellular antigen recognition domain (the immunoglobulin scFv fragment), a flexible hinge, a transmembrane domain, and the intracellular portion that is made of the costimulatory domain (CD28 or 4‐1BB) and the signaling domain (CD3ϛ). The design of this synthetic receptor varies depending on the TAA, and the target TME features. B) CAR designed to improve infiltration can be engineered to I) co‐express chemokine receptors (TGF‐β, IL‐4, CCL‐2, CCL‐19), II) secrete heparinase that degrade the ECM, III) to express bispecific receptors binding the TAA and the FAP presented by CAFs. C) CAR‐T cells designed to remodel the aberrant tumor vasculature I) specific for the VEGFR on endothelial cells, II) the CD248 on pericytes, III) and as switch‐on CAR expression triggered by SynNotch‐P‐selectin D) CARs able to respond to immunosuppressive biochemical inputs include CAR sensitive to i) hypoxia (HIF‐dependent switch ON CAR or a fusion CAR bearing HIFα as signaling domain, II) resistant to oxidative stress (CAR‐T cells with NRF2 knocked out), CAR‐T responsive to acidic pH (CAB‐CAR with scFv portion active only at pH 6.7), and CAR bearing a masking peptide with a linker cleavable by MMPs. E) CAR‐T cells responsive to immunosuppressive cytokines (CAR‐T and IL chimeric receptors co‐expression, SNIPR, armored CAR‐T with an interleukin inducer). F) Engineered cells targeting antigens on immunosuppressive cells include I) TAMs‐binding CAR‐T and II) gene‐modified NK cells bearing a chimeric receptor in which the activating receptor NKG2D is fused to the cytotoxic ζ‐chain of the Tcell receptor (NKG2D.ζ). G) Strategies to revert exhaustion include I) CAR‐T specific PDL1 (check point inhibitory receptors) and FASL (apoptotic signal), II) the coadministration of CAR‐T cells with anti PD1, anti CTLA antibodies already used in clinic, and III) CAR‐T knocked out for the exhaustion regulator TFs (TOX, NR4A2).

Founded at the dawn of 90′,^[^ ^22^ ^]^ synthetic biology has lately become a sophisticated engineering discipline able to orchestrate cellular behavior through the use of genetic circuits that can perform information processing when designed in a sensor‐actuator configuration.^[^ ^23^, ^24^, ^25^ ^]^ Cells modified with synthetic devices found a tremendous application toward the improvement of T cell‐based immunotherapy.^[^ ^3^, ^14^, ^16^ ^]^CARsenable ligand sensing (e.g., cell membrane receptors, glycans, and glycolipids called tumor‐associated antigens (TAA)),^[^ ^16^, ^22^ ^]^ to activate the cytotoxic function and consequently the elimination of the tumor. The CAR is a chimeric single transmembrane receptor constituted of a) an extracellular TAA‐binding domain recognizing domain, b) a flexible hinge, c) a transmembrane domain, and an intracellular T cell signal domain (**Figure** **2**A). Since their first creation by Gross et al., in 1989,^[^ ^26^ ^]^ CAR‐T engineering went through several advancements until reaching a fifth‐generation class. The evolution of CAR‐T structures through generations is already meticulously reviewed by Rafiq et al.^[^ ^7^ ^]^ Despite the anti‐CD19 has been FDA approved for blood malignancies and has improved the clinical outcomes of many patients, some important challenges persist, such as overcoming systemic toxicity, improving efficacy in solid tumors that present the physical barrier of the TME, and antigen heterogeneity. Synthetic biology has employed many strategies to overcome these roadblocks, particularly 1) designing CAR receptor structure to optimize the interaction with the tumor antigen, and 2) inserting genetic circuits able to sense extracellular and intracellular signatures dictated by the TME (**Table** **1**).

**Table 1:** Synthetic biology strategies to engineer CAR‐T to resist the TME.

TME feature	Strategy	Specific target	CAR‐T specificity	Model	Research stage/Clinical validation	Effect	Refs.
Thick ECM	Infiltration‐guiding chemokines	TGF‐β, PSCA, IL‐4	1G.CAR	Human pancreatic ductal adenocarcinoma in mice	Preclinical	Selective antitumor effect on tumor site	[156]
		CCL2	GD2.CAR	Human neuroblastoma xenograft into SCID mice	Preclinical	Improved homing	[30]
		Expressing IL‐17, CCL19	hCD20.CAR	P815‐hCD20 tumor in mouse	Preclinical	Increased infiltration	[32]
	Degrading ECM	Heparanase secretion	GD2.CAR	Melanoma or neuroblastoma model in NSG mouse	Preclinical	Increased infiltration and antitumor activity	[33]
	Attacking CAFs	Targeting FAP	FAP.CAR	Malignant pleural mesothelioma, colorectal carcinoma, lung adenocarcinoma in mice	NCT01722149 Clinical Phase I (completed)	Increased antitumor activity	[34]
	Targeting two antigens of TME	Targeting FAP and GPC3 antigens	FAP‐GPC3.CAR	Hepatocellular carcinoma (NSI mice)	Preclinical	Higher anti‐tumor efficacy compared to single target CAR	[35]
	Attacking CAFs and engaging T cells	Secreting anti‐FAP and anti‐CD3	Mesothelin.CAR	Pancreatic adenocarcinoma in NSG mice	Preclinical	Significant binding to antigens, high antitumor activity	[36]
	Targeting both tumor cells and CAFs	Targeting FAP, SLAMF7	BCMA.CAR	Multiple myeloma with BM‐CAFs in xenograft mouse	Preclinical	Improved effector functions	[37]
Irregular vasculature	Targeting stromal cells	N/A	CD248.CAR	Breast cancer, Lung cancer inmice	Preclinical	Tumor necrosis, reduced tumor size and metastasis	[38]
	Targeting receptors of blood vessels	VEGFR	VEGFR2‐CAR	Metastatic cancer	NCT01218867 (terminated)	No clinical benefit in clinical trials	[40, 43]
	Targeting adhesion protein with SynNotch	P‐selectin	Anti‐GD2.CAR	Neuroblastoma mouse model	Preclinical	Increased anti tumor efficacy	[44]
Hypoxia	Harnessing oxygen availability of TME	HIF‐1a	Pan‐anti‐ErbB targeted CAR	Hypoxic HN3 tumors in NSG mice	Preclinical	Anti‐tumor activity without off‐tumor toxicity	[48]
Oxidative stress	Knockdown of Nrf2	Nrf2	CD19.CAR	Melanoma, lymphoma, colon carcinoma, lung carcinoma in mice	Preclinical	Increase in antitumor activity and survival of CAR‐T cells	[47]
Matxix proteases	Masking CAR	Protease	EGFR.mCAR	Subcutaneous human lung cancer xenograft in NSG mice	Preclinical	Improved safety profile, increased antitumor efficacy	[46]
Immune‐suppressive biochemical signals	Co‐expressing anti‐TAA and chimeric receptors for suppressive cytokines	IL‐4	MUC1.CAR	MDA‐MB‐435, ZR75, MCF7 cell lines, mouse model	Preclinical	MDA‐MB‐435	[53]
	Creating immune‐responsive phenotypes	Secreting IL‐18	CD19.CAR	EL4hCD19+ tumor in syngeneic mice	Preclinical	Enhanced in vivo expansion and persistence of CAR‐T cells	[45]
Immune suppressive cells	Inhibiting TAMs	F4/80	F4.CAR	HKP1 lung tumors in cancer	Preclinical	Increased infiltration, antitumor activity	[63]
E	Inhibiting MDSCc	NKG2D	GD2.CAR, NKG2D.CAR	Neuroblastoma in mice	Preclinical	Improved antitumor compared to CAR‐T therapy alone	[64]
	Targeting T‐r‐eg cells	orthoIL‐2 and orthoIL‐2Rβ	Transgenic CD8+ T cells	Melanoma in mice	Preclinical	Reduced pro‐tumoral Treg cells	[65, 66, 67]
T cell exhaustion	Triple knockout of TFs	Nr4a1, Nr4a2, Nr4a3	huCD19.CAR	Melanoma in mice	Preclinical	Tumor regression, prolonger survival of tumor bearing mice	[71]
	Knockout of transcription factor or checkpoint receptors	TOX, PD1, TGF‐βR	CD19.CAR, Mesothelin CAR, EGFR.CAR	Melanoma in mice	NCT03747965, NCT03545815, NCT04976218 (unknown status in clinical)	Decreased exhaustion in preclinical	[157]
	Co‐expression of immune checkpoint blockade single chain variable fragment (scFv)	PD‐1	CD19.CAR or MUC16.CAR	Peritoneal carcinomatosis, ovarian carcinoma in mice	Preclinical	Improved safety	[158]
	Using Chimeric switch receptor	PDL1	CD19.CAR or mesothelin.CAR or PSCA.CAR	EMMESO flank tumor‐bearing NSG mice, PC3‐PSCA flank tumor bearing mice	Preclinical	Enhanced tumor lytic activity, cytokine secretion	[69]
	Preventing FasL‐induced apopotosis	FasL	CD19.CAR or pmel‐1.CAR	Melanoma in mice	Preclinical	Increased longevity, antitumor activity of T cells	[70]

### 2.1 Synthetic Biology for Designing TME ‐Responsive CAR‐T

One of the challenges of translating CAR‐T cell therapy to solid tumors resides in the complexity of the TME that creates a biochemical and physical barrier that prevents them from infiltrating and eliminating cancer cells^[^ ^9^ ^]^.TME is characterized by immunosuppressive chemokines/cytokines and cells, as well as hypoxia, oxidative stress, acidic pH, and abnormal leaky vasculature (Figure 1).^[^ ^10^ ^]^ Besides hindering the infiltration capacity this pathological context also induces T cell exhaustion, an anergic state where T cells lose their cytotoxic and proliferative capacity.^[^ ^27^ ^]^ Least and less explored, the impact of the biomechanical TME features on CAR‐T cell fitness is another crucial element and comprises increased ECM stiffness and protein deposition, aberrant and leaky vasculature, and fluidic pressure (**Figure** **1**).

#### 2.1.1 Strategies to Improve the Infiltration of CAR‐T in the TME

##### Engineering CAR‐T Responsive to the Infiltration‐Guiding Chemokines

Several strategies have been explored to improve CAR‐T cell infiltration into the TME (Figure 2B). Here, a strong immunosuppressive environment is orchestrated by the cancer cells to neutralize immune cells activity. To counter this, CAR‐T cells have been engineered to express receptors for chemokines commonly overexpressed in solid tumors, such as transforming growth factor (TGF)‐β, chemokine (C‐C motif) ligand (CCL)‐2, and CCL19 (Figure 2BI). For example, a hybrid CAR with the TGFβ receptor's extracellular domain can convert this inhibitory signal into activation (Figure 2BI).^[^ ^28^, ^29^ ^]^ Similarly, CAR‐T cells expressing C‐C chemokine receptor type 2‐(CCR2) target CCL2 enhance infiltration and tumor suppression in models of mesothelioma and neuroblastoma (Figure 2BI).^[^ ^30^, ^31^ ^]^ Co‐expression of interleukin (IL)‐7 and CCL19 in CAR‐T cells has also improved infiltration in solid tumor models (Figure 2BI).^[^ ^32^ ^]^ These approaches reprogram suppressive signals into stimulatory ones but may reduce T cell activation thresholds and increase the risk of off‐target effects.

##### Engineering CAR‐T Cells to Degrade the ECM in the TME

Another strategy to enhance CAR‐T infiltration is by breaking down the ECM, which is denser and more rigid in solid tumors due to the presence of fibrous proteins, glycosaminoglycans, and proteoglycans (Figure 1). Disialoganglioside 2 (GD2)‐CAR‐T cells secreting heparanase have been shown to degrade ECM and improve infiltration in neuroblastoma and melanoma models, boosting survival (Figure 2BII).^[^ ^33^ ^]^ Targeting cancer‐associated fibroblasts (CAFs), which promote ECM thickening, is another approach. CAR‐T cells against fibroblast activation protein α (FAP) have entered clinical trials (NCT01722149), though concerns remain.^[^ ^34^ ^]^ To improve safety and efficacy, bispecific CAR‐T cells targeting both FAP and tumor antigens (e.g., glypican 3 (GPC3) or mesothelin (MSLN)) have shown promise in preclinical models (Figure 2BIII).^[^ ^35^, ^36^ ^]^

Additionally, SLAMF7‐CAR‐T cells can target both CAFs and multiple myeloma cells,^[^ ^37^ ^]^ though SLAMF7's expression on other lymphoid cells raises off‐target toxicity concerns. Targeting endosialin (CD248), found on stromal cells in tumor vasculature, also showed reduced tumor growth and metastasis in mouse models, making it a potential new target (Figure 2CII).^[^ ^38^ ^]^

#### 2.1.2 Engineering CAR‐T to Remodel Cancer Blood Vessels

Abnormal tumor vasculature is characterized by leaky vessels, high pressure, and hypoxia(Figure 1) hinders CAR‐T cell infiltration.^[^ ^39^ ^]^ To address this, CAR‐T cells targeting vascular endothelial growth factor receptors (VEGFR)s, overexpressed in tumor vessels, have been developed (Figure 2CI), showing tumor suppression in various xenograft models.^[^ ^40^, ^41^, ^42^ ^]^ However, a phase I/II trial of VEGFR2‐CAR‐T for metastatic cancer showed no clinical benefit (NCT01218867), due to VEGF‐A overexpression outcompeting CAR binding.^[^ ^40^, ^43^ ^]^ Additionally, VEGF pathways are active in normal tissues, raising concerns about off‐tumor toxicity. However, a recent study sheds hope on efficiently targeting the TME vasculature with a switch‐on synthetic circuit, avoiding toxicity. Vogt et al., developed a synthetic Notch (synNotch) receptor that selectively expresses an anti‐GD2‐CAR (Neuroblastoma antigen) upon binding to P‐selectin, a cell adhesion protein overexpressed in tumor new blood vessels.^[^ ^44^ ^]^ These cells induced a significant reduction of tumor volume and survival over 21 days in a neuroblastoma mouse model, highlighting the antitumoral potential of TME‐based sensors (Figure 2CIII).

#### 2.1.3 Engineering CAR‐T to Exploit and Remodel the Immune Suppressive Biochemical Cues of the TME

Beyond improving infiltration, CAR‐T cells have been engineered to sense and respond to TME‐specific biochemical cues to enhance tumor specificity and reduce off‐target toxicity. These include hypoxia‐, reactive oxygen species (ROS), and pH‐responsive CARs, as well as protease‐activated and cytokine‐sensing designs (Figure 2D).^[^ ^45^, ^46^, ^47^, ^48^, ^49^ ^]^

##### Engineering CAR‐T Cells to Respond to Hypoxia

Hypoxia‐sensitive CAR‐Ts were found to exhibit a specific antitumor effect in some carcinoma xenografts.^[^ ^49^, ^50^ ^]^ Kosti et al., developed an on/off oxygen‐sensing switch attached to receptor tyrosin kinase ErbB‐CAR‐T. In shortage of oxygen, the hypoxia induceble factor (HIF)α binds the sensor and induces CAR expression (Figure 2DI). These cells that achieved specific tumor elimination without off‐tumor toxicity.^[^ ^50^ ^]^

In a more recent study, the same HIFα sensing domain was fused to a CAR‐T specific for the carcinoembryonic antigen (CEA), obtaining also here a CAR‐T activation highly restricted to the hypoxic tumor environment (Figure 2DI).^[^ ^49^ ^]^ This approach can minimize the off‐target cytotoxicity as the CAR stops functioning in a normoxic environment. TME is also enriched in ROS that undermine the T cell cytotoxic function through the nuclear factor erythroid 2‐related 2 (Nrf2). Last year Jo et al., demonstrated that the knock‐down of Nrf2 (Figure 2DII) reinforced TILs and CD19‐CAR‐T cell antitumor activity in vivo, shedding light on Nrf2 as a new therapeutic target.^[^ ^51^ ^]^

##### Engineering CAR‐T Cells Sensitive to Acidic pH

The TME also presents acidic pH, which is due to the increase of glycolysis rate in cancer cells and favors immune escape and inhibits the T cell growth.^[^ ^52^ ^]^ CAR‐T able to convert the immune suppressive pH input into activation signals are the anti‐AXL receptor tyrosine kinase (CCT301‐38) and the anti‐ receptor tyrosine kinase‐like orphan receptor‐(ROR)2 (CCT301‐59), currently in clinical trials (NCT03393936). These CAR‐T are termed Conditionally Active Biologics (CAB)‐CAR‐T (Figure 2DIII) as their CAR receptor bears a particular single‐chain variable fragment (scFvs) domain that becomes activated only in response to the acidic conditions (pH∼6.7) within TME, therefore increasing tumor‐specificity and reducing on‐target off‐tumor toxicity through a reversible “AND” boolean logic gate.

##### Engineering CAR‐T Cells Sensitive to Matrix Proteases

As the TME also brims of matrix proteases to facilitate invasion, another study generated a CAR‐T where the antigen‐binding site is masked with a protease‐responsive domain. In this way, the CAR can recognize the TAA only upon the cleavage of the peptide, therefore activating the cells only in the TME and avoiding non‐specific action on healthy tissue (Figure 2DIV).^[^ ^46^ ^]^

##### Engineering CAR‐T Cells IL‐Responsive

Chimeric receptors presenting IL‐4 receptor (IL‐4R) extracellular portion fused to either the cytoplasmic part of the IL‐2 receptor (IL‐2Rβ)^[^ ^53^ ^]^ or the inner domain of the IL‐7 receptor (IL‐7R) (Figure 2DV )^[^ ^54^ ^]^ co‐expressed with anti‐ prostate stem cell antigen (PSCA)‐CAR, strongly elicited T cell accumulation and cytotoxicity in vitro.^[^ ^53^, ^54^ ^]^ Sukuraman et al., engineered anti‐ PSCA CAR T cells with cytokine‐specific chimeric receptors such as the IL‐4R– IL‐7R and a TGF‐βR–4‐1BB (Figure 2DV), that were capable of effectively eliminating the tumors where IL‐4 and TGF‐β were highly expressed.^[^ ^29^ ^]^ Trying to shift the balance of TME from immunosuppressive to a favorable immune‐responsive phenotype is another challenge that have been engaged, specifically with armored CAR‐T engineered to secrete pro‐inflammatory cytokines such as IL‐12,^[^ ^55^ ^]^ IL‐18,^[^ ^45^ ^]^ and IL‐23 (Figure 2DV).^[^ ^56^ ^]^

Engineered T cells to present a subunit of IL‐23 which can reconstitute into a functional IL‐23 in TME, can enhance T cell proliferation, and minimize off‐target cytotoxicity.^[^ ^45^ ^]^ CAR‐T cells releasing IL‐12 or IL‐2 can attract in site pro‐inflammatory M1 macrophages, while the autocrine signals on T cells induce the production of interferon‐γ (IFN‐γ), which suppresses T regulatory lymphocytes(T‐reg) which inhibit CAR‐T.^[^ ^45^, ^55^, ^57^, ^58^ ^]^

Taken together, these studies suggest that reshaping the TME through secreting antitumor cytokines could be a winning strategy in the CAR‐T fight against solid tumors. However, it is critical to control their expression and release to avoid systemic toxicities.

#### 2.1.4 Engineering CAR‐T to Inhibit the Immune‐Suppressor Cell Types of TME

As aforementioned, the TME also abounds of dysregulated cells that exacerbate the immune‐suppression and correlate with relapses and toxicity to CAR‐T therapy (Figure 1).^[^ ^59^, ^60^ ^]^ Therefore, besides targeting the immune suppressive cytokines, another approach of synthetic biology consists in directly attacking the secreting cells, such as the myeloid cells, and T‐regs.^[^ ^61^ ^]^ The flourishing attention of the suppressive role of the myeloid niche of the TME^[^ ^62^ ^]^ has driven efforts in targeting tumor associated macrophages (TAM) and myeloid‐derived suppressive cells (MDSCs) (Figure 1, 2F).

CAR‐T against TAMs, expressing the F4.CAR (specific for the macrophage F4/80) has been found to reduce growth and prolong survival in lung, ovarian, and pancreatic murine models (Figure 2Fi). Although not directly targeting the tumor, the F4.CAR eliminated the macrophages and increased the endogenous CD8 T cell portion through the secretion of IFN‐γ that as mentioned before, inhibits T‐reg.^[^ ^63^ ^]^

Parihar et al., developed NK cells expressing a receptor fusing the NK cytotoxic outer (NKGD2) domain with the CAR‐T inner portion, able to kill MDSCs in a neuroblastoma xenograft model (Figure 2Fii). Furthermore, NKG2D‐CAR NK cells prompted CAR T cell action when co‐administered with disialoganglioside (GD2)‐neuroblastoma TAA‐ CART cells, suggesting the clinical potential of a combinatorial therapy that paves the way to CAR‐T cell by eliminating the MDSC‐mediated immunosuppressive inputs.^[^ ^64^ ^]^

T‐reg cells in the TME hinder CAR‐T efficacy by competing for IL‐2 via their high IL‐2Rα expression. IL‐2 secretion or administration may unintentionally favor T‐reg expansion. To avoid this, engineered IL‐2 variants that selectively bind IL‐2Rβ on CAR‐T cells have been developed. A more precise solution uses orthogonal IL‐2/IL‐2Rβ pairs (orthoIL‐2/orthoIL‐2Rβ), promoting selective CAR‐T proliferation while minimizing T‐programmed death protein 1reg activation.^[^ ^65^, ^66^, ^67^ ^]^

This research highlights how insidious it is to unleash the CAR‐T in the solid TME, as the factors that drive immunosuppression are multiple and intricate. Combinatorial strategies that aim at remodeling the TME in diverse aspects are challenging and needed.

#### 2.1.5 Engineering CAR‐T for Preventing Cell Exhaustion

As mentioned above, one of the reasons for the inefficiency of CAR‐T in solid tumors is the onset of exhaustion upon chronic antigen stimulation and other stimuli imparted by the TME (Figure 2G). One feature of this anergic state is the upregulation of the inhibitory checkpoint receptotrs that mediate immune escape (e.g programmed death protein 1 (PD1), cytotoxic T‐lymphocyte antigen 4 (CTLA‐4)).^[^ ^68^ ^]^ An approach to counteract exhaustion is to convert inhibitory and apoptotic signals into a survival cell response. This has been achieved by equipping CAR‐ T cells with chimeric PD1^[^ ^69^ ^]^ and dead signaling FAS (Figure 2GI),^[^ ^70^ ^]^ presenting a modified intracellular moiety that leads to the activated effector phenotype. In another study now under clinical trial (NCT04577326), mesothelin‐CAR‐T bearing also the PD1 dominant negative receptor (DNR) were observed to have increased activity in a xenograft model of pleural mesothelioma presenting high levels of the PD1 ligand (PDL1) (Figure 2G).^[^ ^69^ ^]^ The advantage of expressing the PD1 chimeric antigen compared to the standard coadministration of PD1 inhibitors (Figure 2GII), lies in avoiding repeated doses with the same positive outcome.^[^ ^69^ ^]^ Among the transcription factors that drive exhaustion, NR4A2^[^ ^71^ ^]^ and thymocyte selection‐associated high mobility group box protein (TOX),^[^ ^72^ ^]^ which are crucial mediators of epigenetic and transcriptional modification, have been involved in genetic engineering strategies to counteract exhaustion (Figure 2GIII).^[^ ^73^, ^74^ ^]^

## 3 The Synergy Between Synthetic Biology and Biomaterials to Improve CAR‐T Efficacy Against TME

**Figure 3:**
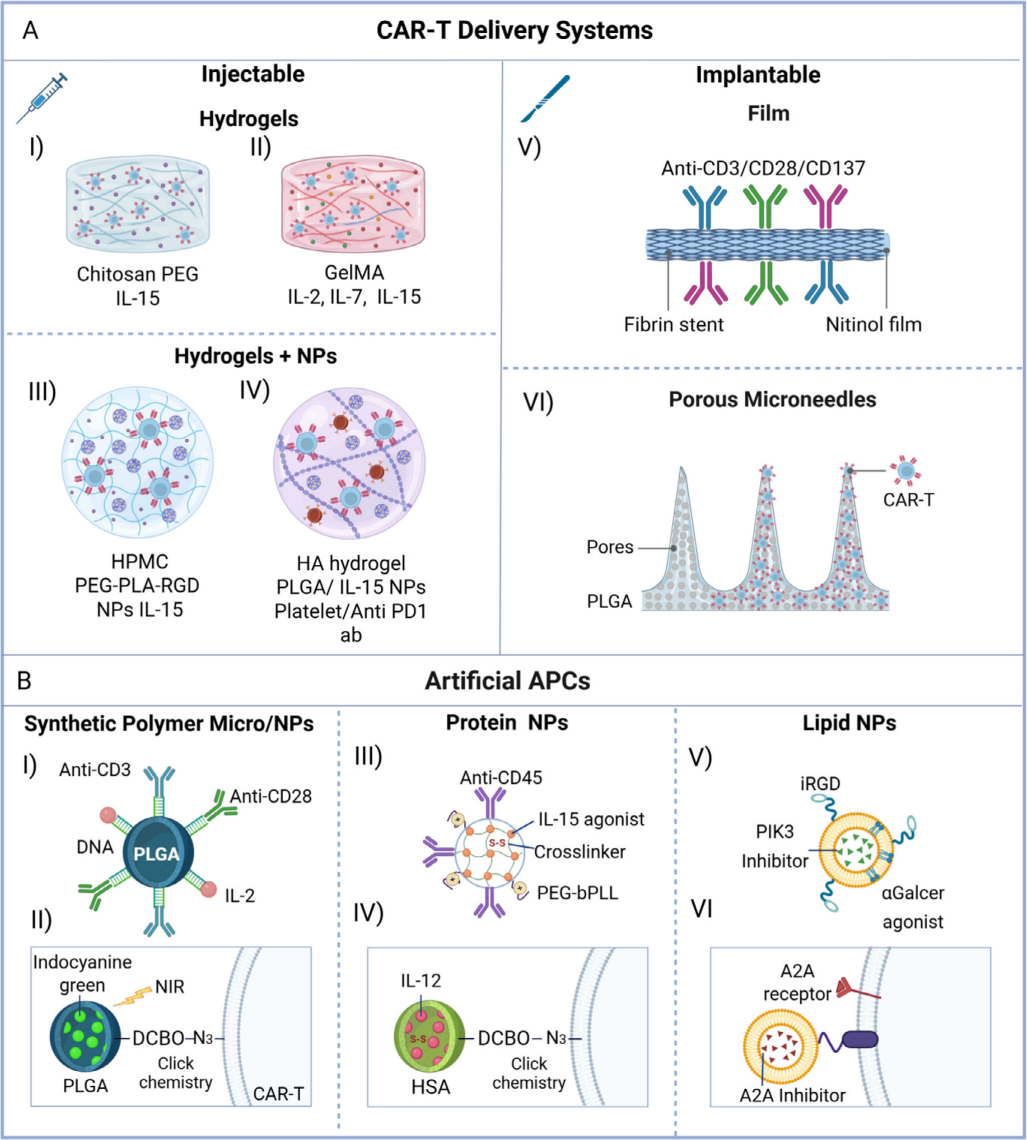
Schematic representation of biomaterials used as delivery systems and artificial APCs for CAR‐T therapy. A) Delivery systems are I‐IV) injectable or V‐VI) implantable at the tumor site. Injectable systems include hydrogels based on I) Chitosan‐PEG or II) GelMA loaded with CAR‐T‐guiding cytokines, and hydrogels based on III) HPMC or IV) HA containing NPs (PEG‐PLA‐RGD, PLGA or platelet). NPs usually bear a cytokine cargo that directs CAR‐T cells. CAR‐T can also be delivered in situ through V) implantable nitinol‐fibrin films (functionalized with the binding‐activation antibodies (anti‐CD3, anti‐CD28, and anti‐CD137) and VI) through PLGA patches with porous microneedles. B) Micro/nanoparticles not only function as delivery systems, but also as artificial antigen presenting cells (APCs) for CAR‐T. APCs can be synthetic polymers I, II) NPs PLGA, III, IV), protein NPs (HSA) and V, VI) NPs can be functionalized with activation antibodies (anti‐CD3, anti‐CD28, and anti‐CD45) or cell adhesion peptides (iRGD). They can bear cargo cytokines (IL‐2, IL‐12), agonists (Il‐15 and αGalcer) or inhibitors (PIK3, A2A) that support CAR‐T function. In some cases the NPs can be conjugated to the CAR‐T through click chemistry or through fusion to a transmembrane protein. Figure created with biorender.com.

**Figure 4:**
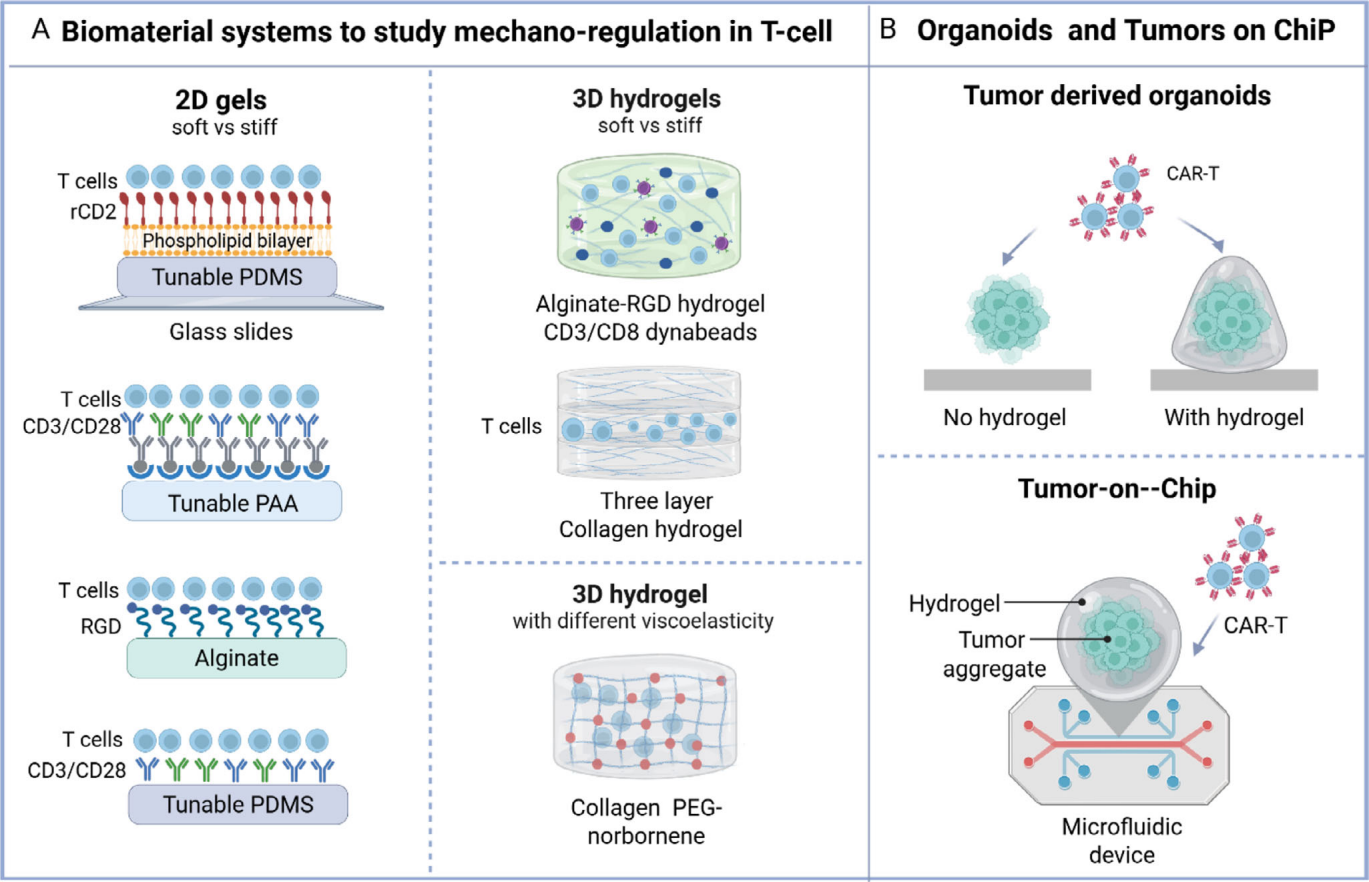
Biomaterial systems, organoids, and microfluidic devices as platforms for mechanistic studies and CAR‐T efficacy screens. A) 2D and 3D gels mimicking the ECM stiffness effect on T cells used to evaluate T cell activation, proliferation, phenotype, and exhaustion. B) Tumor organoids and tumor on chips as ex vivo comprehensive platform of the cellular, biochemical the physical (stiffness, fluid pressure and microarchitecture) TME elements that have been used to evaluate infiltration, activation, killing capacity, antigen recognition, and effector cytokine release. Figure created with biorender.com.

Despite all these efforts from synthetic biology, the challenge posed by the TME requires a multidisciplinary and synergistic approach to reach therapeutic efficacy in solid tumors. Biomaterials have crossed the CAR‐T path in many ways: 1) as tools to enhance the CAR transduction efficiency 2) as in vitro platforms for CAR‐T cell expansion prior administration, 3) as a delivery system for CAR‐T in situ, 4) as artificial APCs, 5) in 2–3D system/organoids and microfluidic devices as platforms for mechanistic studies and CAR‐T efficacy screen. The first two in vitro applications have been thoroughly reviewed somewhere else,^[^ ^12^, ^75^, ^76^ ^]^ therefore, this chapter is first focused on the last two in vivo applications that have the purpose of paving the way of CAR‐T through the solid TME (Sections 3.1‐3, **Figure** **3**, **Table** **2**).^[^ ^77^ ^]^ Second, the application of biomaterials in 2D‐3D systems and organoids/ microfluidic devices as screening platforms for CAR‐T will be summarized (Sections 3.3, **Figure** **4**, **Table** **3**).

**Table 2:** Biomaterials as delivery systems and APCs.

System	Material	Functionalization/Enacapsulation	CAR‐T	Model	Effect	Refs.
Hydrogel	Chitosan‐PEG	IL‐15	GD‐2.CAR‐T	Retinoblastoma, mouse model (In situ xenograft nude mice model)	Prevents tumor recurrence	[79]
	GelMA	IL‐2, IL‐7 and IL‐15	HER2. CAR	Melanoma, mice	Improves anti‐tumor efficacy in both in vitro and in vivo conditions	[80]
Hydrogel+ nanoparticle	HPMC‐C12 and PEG‐PLA nanoparticles	IL‐15	B7H3‐ specific CAR	Subcutaneous human medulloblastoma (MED8A) in NSG mice	Enhances sustained survival, functionality and activation of CAR‐T cells	[78]
	HA, PLGA nanoparticles	IL‐15, anti‐PDL1‐conjugated platelets	CSPG4.CAR	Subcutaneous melanoma in NSG mice	Enhances T‐cell persistence and anti‐tumor activity	[82]
Micromesh	Nitinol, fibrin	Anti‐CD3, Anti‐CD28 and Anti‐CD137	N/A	Non‐resectable ovarian cancer, mice model	Enhances expansion of T cells and direct delivery of T cells to the tumor	[84]
Nano/micr‐oparticles	Silica, alginate	Cyclic di‐GMP, anti‐CD3, anti‐CD28, anti‐CD137	NKG2D.CAR	Subcutaneous melanoma model, orthotopic mouse model of pancreatic cancer	High expansion of CAR T cells in tumor site	[88]
	PLGA, DNA scaffolds, PEG, PLA	Anti‐CD3, Anti‐CD28, IL‐2	HER2.CAR	Leukemia cell line K562 xenograft tumor in NSG mice	Presentation of priming antigens and efficient activation of CAR‐T cells	[94]
	HSA, GSH, DBCO	IL‐12	CD19.CAR	NOD‐SCID mice injected with Raji‐luc cells	Selective recruitment and enhanced expansion of CAR‐T cells in solid tumor	[95]
	PEG, NHS‐SS‐NHS crosslinker	Il‐15Sa, Anti‐CD45 ab, Glutathione	EGFR.CAR	B16F10 melanoma mouse model, U‐87 MG human glioblastoma in NSG mice	Supports cell expansion	[159]
	Cross‐linked liposomal vesicles, maleimide‐functionalized lipids	SCH‐58261	Anti‐CD19 CAR	SKOV3.CD19 ovarian cancer model and K562.CD19 leukemia model in NSG mice	Prevents CAR‐T cells hypofunction	[97]
	Lipid nanoparticles (DSPE‐PEG‐maleimide)	iRGD, PI3K inhibitors, alpha‐GalCer agonist	Anti‐ROR1 CAR	4T1 syngeneic breast cancer model and human glioma in mice	Tumor localization signal	[98]
	PLGA	Indocynine green	Anti‐CD19	Burkit lymphoma model	ECM loosening agent	[160]
INPs, Magnetic control	Iron oxide nanoparticles (SPIONs)	SPIONs	CSPG4.CAR	In vitro model of Melanoma expressing chondroitin sulfate proteoglycan	CAR‐T cell localization to tumor, Avoids off tumor toxicity	[101]
INPs, PPT	Smart gold (sAuNPs)		OT‐1 transgenic T cells	Melanoma murine model (F10‐OVA)	Efficient infiltration	[102]
	Gold	Polydopamine, horseradish peroxidase (HRP)		NALM 6 solid murine model	Tumor elimination, persistent immune memory cells	[104]
	Au/Cu_2‐_ _x_Se and 3‐bromopyruvate	Camouflaged with CAR‐T membrane	CD19.CAR	NALM6 solid tumor	Tumor elimination	[105]
	Chromium	Polydopamine	B7‐H3 CAR‐T	Hepatocarcinoma and breast tumor bearing mice	Improvement of CAR‐T cell infiltration and tumor growth inhibition	[106]

**Table 3:** Biomaterials for ECM‐mimicking mechanistic studies on T cells.

Dimension	Biomechanics	Biomaterial	Functionalization	Method	T‐cell	Effect	Refs.
2D	Stiffness	PDMS	Anti‐CD3, Anti‐CD28	Adsorption	CD8+	Mechanical stress supports CD8+ T‐cell exhaustion	[114]
		PDMS	Anti‐CD3, Anti‐CD28	Silanization	CD4+	Softer substrate supports CD4+ T‐cell expansion	[161]
		PDMS	rCD2	Lipid bilayer formation on PDMS	Jurkat	Early TCR triggering is not mechanosensitive	[131]
		PAA	Anti‐CD3, Anti‐CD28, biotinylated antibody	Biotin‐streptavidin binding	CD4+/CD8+	Ligand density and intermediate stiffness induces T‐cell activation	[133]
		Alginate	GP 61–80 peptide	Conjugation of RGD peptide to alginate	CD4+	Stiffness suppresses proliferation via YAP induction	[132]
3D	Stiffness, Density	Collagen hydrogel	N/A	Encapsulation	TILs	Reduction of T‐cell proliferation in high density matrix and vice versa	[124]
	Viscoelasticity	Collagen‐PEG‐Norbonene hydrogel	N/A	Click‐chemistry	CD8+	Role of matrix viscoelasticity of generating distinct T‐cell phenotypes	[21]

### 3.1 Biomaterial‐Based Constructs as CAR‐T Delivery System In Vivo

The gold‐standard CAR‐T endovenous intravenous administration suffers poor penetration, growth, and scarce anticancer activity in the solid tumor site.^[^ ^16^, ^78^, ^79^, ^80^ ^]^ Biocompatible materials have demonstrated the ability to continuously deliver functional immune cells into tumors.^[^ ^81^ ^]^ Therefore, biomaterials‐based in situ delivery systems can aid CAR‐T cells by overcoming physical barriers and immune‐modulate TME, thus promoting successful local CAR‐T cell therapy for solid malignancies.^[^ ^75^, ^76^ ^]^ With this purpose, a variety of biocompatible biomaterials have been engineered to 1) create a protective and supportive niche for CAR‐T expansion and 2) offer a controlled and prolonged release of cytokines and CAR‐T at the tumor site.^[^ ^82^, ^83^ ^]^ Specifically, biomaterial constructs such as hydrogels alone or in combination with organic, inorganic or hybrid nano/micro‐particles, film, and patches (Figure 3A) have been found to improve CAR‐T retention/activity in several cancer preclinical models such as retinoblastoma, medulloblastoma, melanoma, glioblastoma, breast, ovarian, and pancreatic cancer (Table 2).^[^ ^84^, ^85^, ^86^, ^87^, ^88^ ^]^ The innovative design of several of these constructs reside into taking in account the TME features to improve CAR‐T cell proliferation, activation and tumor killing capacity (Figure 3A). For example, Han's group,^[^ ^79^ ^]^ engineered an injectable hydrogel to deliver GD2‐specific CAR‐T to treat retinoblastoma. The system, made in a chitosan‐polyethylene glycol (PEG) thermosensitive polymer, not only contains CAR‐T cells but also a stimulatory signal such as IL‐15 that induces expansion (Figure 3AI). GD2‐CAR‐T cells retained their cytotoxic activity, eliminated retinoblastoma, persisted up to 60 days, and structural recovery of retina and vision was also observed in a mouse model. Also, Zhou et al., developed an injectable hydrogel for in situ CAR‐T cell administration. This system, based on the photocross‐linkable gelatin methacryloyl (GelMA), was loaded with even more T cell growth cytokines such as IL‐2, IL‐7, and IL‐15 (Figure 3AII). Crosslinked with blue light after injection, the GelMA degraded in 14 days, ensuring stable release and extended the permanence of the CAR‐T in the TME and therefore the anti‐melanoma efficiency.^[^ ^80^ ^]^

Some authors combined hydrogels with nanoparticles (NPs) to maximize the reservoir potential and obtained encouraging results in some tumor models (Figure 3AIII‐IV).^[^ ^78^, ^82^ ^]^ For instance, a recent study presented an injectable hydrogel made of ‐hydroxypropyl methylcellulose (HPMC) polymer mixed with NPs poly PEG‐b‐poly (lactic acid) (PEG‐PLA) NPs and functionalized the cell‐binding peptide arginine‐glycine‐aspartic acid (RGD). The system was encapsulated with B7H3‐ specific CAR‐T cells and the stimulatory cytokine IL‐15 (Figure 3AIII), thus creating an inflammatory niche for controlled expansion (100‐fold) and release of CAR‐T cells that resulted in the suppression of medulloblastoma solid tumor in 100% of mice within 21 days.^[^ ^78^ ^]^

Hu et al.^[^ ^79^ ^]^ developed a hydrogel system combining IL‐15‐loaded poly‐lactic‐co‐glycolic acid (PLGA) NPs and platelet/anti‐PD‐L1 components within a hyaluronic acid (HA) matrix to enhance CAR‐T activity against chondroitin sulfate proteoglycan 4 (CSPG4) in melanoma (Figure 3AIII). The biocompatible HA matrix degraded in vivo after 8 days, ensuring sustained‐release of CAR‐T that resulted in significantly reduced tumor mass (60‐fold) in mice (Figure 3AIV) after 3 weeks. CAR‐T persisted in the tissue for 4 weeks. Scaffolds also show promise for delivering CAR‐T cells to eliminate residual or inoperable tumors. Coon et al. created a fibrin‐coated nitinol micromesh functionalized with anti‐CD3, CD28, and CD137 to prevent ovarian cancer recurrence (Figure 3AV),^[^ ^84^ ^]^ and later developed a silica‐based scaffold loaded with CAR‐T cells and an IFN gene agonist for treating pancreatic cancer.^[^ ^88^ ^]^

An alternative method proposed by Li et al developed a PLGA porous microneedle patch loaded with CAR‐T cells that has been implanted subcutaneously in a mouse melanoma mouse model after post‐surgical resection of the 90% of the tumor (Figure 3AVI). This ensured a scattered delivery and systemic distribution of CAR‐T cells that resulted in the elimination of the residual tumor and recurrence prevention.^[^ ^87^ ^]^

I Taken together, these preclinical results suggest that hydrogel or film‐based constructs hold great promise for the translation of CAR‐T to solid tumor immunotherapy. The potential of this therapeutic approach lies in the possibility of incorporating costimulatory co‐factors and exposing the tumor with a higher concentration of immune cells over a prolonged timeframe. Furthermore, the local presentation can increase the safety of therapeutic agents, as biomaterials also offer fine‐tunable support for surface functionalization, which is detrimental for modulating immune cell performance. Nevertheless, scaling up delivery systems still represents significant challenges like assuring reproducibility in mechanical strength during, degradation rate, biocompatibility, sterility, and safety. Computational modeling to predict optimal formulations and 3D printing hold the potential to standardize hydrogel fabrication in a large scale.^[^ ^89^ ^]^ Moreover, standardized international guidelines are needed to reach regulatory approval.

### 3.2 Biomaterials Such as Nano/Microparticles to Improve Antigen Presentations to CAR‐T in the TME and Delivery

#### 3.2.1 Organic Nano/ Microparticle as Artificial APCs

The immunosuppressive TME not only impedes CAR‐T infiltration but also disrupts activation and function, which rely on APCs.^[^ ^86^ ^]^ While CARs deliver antigen and costimulatory signals, cytokines like IL‐2, IL‐7, and IL‐15 normally provided by APCs—are often lacking in the TME, where tumor cells dominate and suppress immunity.^[^ ^85^, ^86^, ^90^ ^]^ Biomaterials can mimic APCs by releasing cytokines and presenting costimulatory signals to enhance CAR‐T activation and proliferation.^[^ ^91^, ^92^, ^93^ ^]^ Various organic nano/microparticles, made from polymers, proteins, and lipids, have been used as synthetic APCs, boosting CAR‐T efficacy in preclinical cancer models (Figure 3B) (Table 2).^[^ ^12^, ^77^ ^]^

Desai's group. developed DNA–PLGA microparticles (∼2 µm) decorated with CD3/CD8 antibodies and IL‐2, which, when injected into leukemia‐bearing mice, attracted systemically delivered CAR‐T cells and led to tumor clearance (Figure 3Bi).^[^ ^77^, ^94^ ^]^ Photothermal PLGA nanoparticles loaded with indocyanine green and attached to anti‐CD19 CAR‐T cells enhanced tumor penetration upon near infrared (NIR) stimulation by loosening the ECM, improving blood flow, and suppressing Burkitt lymphoma (Figure 3BII). Another approach used IL‐12‐loaded human serum albumin (HSA) NPs linked to CAR‐T cells via click chemistry. Triggered by antigen engagement, these NPs released IL‐12 locally, enhancing lymphoma control (Figure 3BII).^[^ ^95^ ^]^ Similarly, Tang et al. created disulfide‐crosslinked protein nanogels that surrounded CAR‐T cell membrane and systemically injected into a murine melanoma model. The unit is a nanoparticle functionalized with an IL‐15 agonist, an anti‐CD45 antibody, and polyethylene glycol‐b‐polylysine (PEG‐b‐PLL) positively charged to interact with the negatively charged cell membrane (Figure 3BIV). This nanogel induced IL‐15 release, leading to CAR‐T expansion and survival of 80% of the mice over 60 days (Figure 3BIII).^[^ ^96^ ^]^ In summary, CAR‐T linked with cytokine‐loaded NPs favored tumor clearance in vivo, suggesting the potential of this approach and that further validations at the clinical level are worthy to be performed.

Following the same purpose, CAR‐T cell membrane has also been functionalized with lipid NPs loaded with some key immune‐stimulant molecules. For example, CD19‐CAR‐T cells conjugated with lipidNPs containing the A2 adenosine receptor (a T cell suppressor) antagonist SCH‐58261 (Figure 3BVI) were found to ameliorate CAR‐T cell function in the TME of the xenograft model.^[^ ^97^ ^]^

Some other lipid NPs were not only used as cargo but also presented a guiding signal for specific tumor localization (Figure 3BV).^[^ ^98^ ^]^ These particles made with the lipid‐ 1,2‐distearoyl‐sn‐glycero‐3‐phosphoethanolamine (DSPE)‐PEG‐maleimide were loaded with phosphoinositide 3‐kinase (PI3K) PI‐3065 inhibitor of immune suppressive cells, and the α‐galactosyl ceramide (α‐GalCer) 7DW8 agonist with anti‐tumor function. They also presented on the surface the internalizing (i)RGD peptide, that binds αv integrin, the target on cancer cells (Figure 3BV).^[^ ^98^ ^]^ When injected in an in vivo model of breast cancer, these particles boosted the tumor homing thanks to the iRGD tag and switched the TME from immune‐suppressive to immune‐stimulating thanks to the released agents that created a room for CAR‐T for eliminating cancer cells.

In summary, organic NPs have been shown to boost CAR‐T cell therapy efficacy by improving the antigen presentation in the TME both by acting as artificial APCs and by remodeling the TME. The results in vivo encourage the validation of these complex fine‐tunable systems at the clinical level. Multiple parameters such as size, shape, stiffness, viscoelasticity, ligand density, and cellular contact zone need to be optimized in a larger scale for moving to clinical trials and commercialization.^[^ ^99^ ^]^

#### 3.2.2 Inorganic Nanoparticles (INP)s and Photothermal Therapy (PPT) Synergy to Boost CAR‐T Efficacy

Several INPs in combination with PPT have been shown to potentiate CAR‐T delivery and efficacy in treating solid tumors. Their biocompatible nature and their capability to adsorb NIR light and converted into heat, has been exploited to guide and fine‐tune CAR‐T activity against solid tumor in several preclinical models (Table 2).^[^ ^100^ ^]^

Earlier this year, Pfister et al., functionalized CAR‐T cells with magnetically controllable iron oxide nanoparticles (SPIONs), to direct them into the tumor site and therefore avoid off‐tumor toxicity. The SPIONs specifically attacked melanoma cells expressing CSPG4.^[^ ^101^ ^]^

Gold nanoparticles (sAuNPs), ‐carrying T cells successfully infiltrate into tumor tissues (20%) and in combination with photothermal stimulation and showed complete tumor eradication after 21 days in a melanoma murine model (F10‐OVA) and furthermore as well as a survival rate of 100%.^[^ ^102^ ^]^

Despite many INPs are under clinical trials as drug depots,^[^ ^103^ ^]^ still some concerns about toxicity, size, uptake, and stability remain.^[^ ^100^ ^]^ Incorporating INPs with CAR‐T membrane is one strategy for increasing their biocompatibility and decreasing toxicity, and avoiding immune reaction.

Indeed, Li, et al., developed a membrane‐coated multifunctional catalyst to improve tumor elimination. This consisted in encapsulating Ag_2_S quantum dots and horseradish peroxidase (HRP)‐loaded Au/polydopamine nanoparticles (Au/PDA NPs) within CAR‐T cell membranes. When administered into a lymphoma (NALM) 6 solid murine model and in combination with both sonodynamic therapy and PPT, the tumor was eliminated, and persistent immune memory cells were observed.^[^ ^104^ ^]^

Another example of this synergetic strategy is provided by Wu et al., that developed Au/Cu_2‐_ _x_Se and 3‐bromopyruvate nanoparticles camouflaged with CD‐19 CAR‐T cell membrane. These bionanohybrids, when coadministered with regular CAR‐T cells and upon NIR stimulation, proved augmented efficacy in eliminating lymphoma (NALM 6) solid tumor mass.^[^ ^105^ ^]^

Another method to address INPs potential toxicity consists in functionalizing nanoparticles with biocompatible layers as demonstrated by Zou et al.^[^ ^106^ ^]^ A nanocomposite made of biodegradable polydopamine as a carrier for chromium nanoparticles (Cr NPs) was co‐ administered with B7‐H3‐CAR‐T cells, in hepatocarcinoma and breast tumor bearing mice. Upon NIR stimulation, the increase of C‐X‐C motif chemokine ligand 13 (CXCL13) and CCL3 chemokine induced by the nanocomposite degradation products generated a tertiary lymphoid structure within the tumor that secreted proliferative cytokines IL‐2, IFN‐γ, and TNF‐α improving CAR‐T cell infiltration and tumor growth inhibition for 16 days.^[^ ^106^ ^]^

Another important application of metallic INPs is in vivo CAR‐T tracking through magnetic resonance imaging and photoacoustic tomography. Nevertheless, this topic goes beyond the scope of this review, and it is exhaustively discussed elsewhere.^[^ ^100^, ^107^ ^]^

### 3.3 Biomaterial Systems, Organoids, and Microfluidic Devices as Platforms for Mechanistic Studies and CAR‐T Efficacy Screens

Despite advances in cell and biomaterial engineering, CAR‐T therapy remains ineffective in solid tumors, partly due to the biomechanical barriers of the TME. Key mechanical challenges include solid stress from tumor growth, interstitial fluid pressure, ECM stiffening from crosslinking, and abnormal tissue architecture (Figure 1).^[^ ^108^ ^]^

It is well‐known that when T cells interact with APCs or cancer cells, through the formation of immunological synapsis, and are subsequently subjected to tensile and traction forces, generated by membrane protrusions or retractions that result in cell spreading and activation through actomyosin cytoskeleton remodeling.^[^ ^109^, ^110^, ^111^ ^]^ Less known are the dynamics of the TME‐mediated mechanical inputs on T cells. Overall, the main mechano‐pathways in T cell are mediated by TCR, calcium influx through piezo channels, integrin signaling, Yes‐Associated Protein (YAP) and transcriptional co‐activator with PDZ‐binding motif (TAZ) and cytoskeleton remodeling.^[^ ^112^ ^]^

The TME mechanical inputs can impair CAR‐T infiltration and promote exhaustion.^[^ ^113^, ^114^ ^]^ While mechanotransduction in cancer has been widely studied,^[^ ^115^, ^116^ ^]^ in vivo models are limited in capturing these dynamics. As a result, ex vivo platforms such as organoids, microfluidics, and tumor‐on‐chip systems have been developed to mimic and fine‐tune TME mechanics, offering insights into tumor growth, therapy resistance, and immune exclusion.^[^ ^114^, ^117^, ^118^, ^119^, ^120^, ^121^ ^]^ However, the impact of mechano‐TME on T and CAR‐T cells remains largely unexplored.^[^ ^122^ ^]^

#### 3.3.1 Biomaterial‐Systems to Evaluate the Impacts CAR‐T Function of TME Mechanical Features

Nowadays, several reports have used 2D and 3D gels of different materials to investigate the impact of the ECM mechanical features both on cancer and T‐cell,^[^ ^21^, ^114^, ^123^, ^124^, ^125^, ^126^, ^127^, ^128^, ^129^ ^]^ but not many on CAR‐T cells yet^[^ ^130^ ^]^ (**Figure** **4**A) (Table 3). Some recent examples are discussed hereafter. Some 2D models, consisting of polydimethylsiloxane (PDMS), polyacrylamide (PAA), and alginate gels, have been useful in elucidating the influence of substrate stiffness on T‐cell activation, proliferation, and functionality (Figure 4A).^[^ ^131^, ^132^, ^133^, ^134^ ^]^ First, Lippert et al., described T cell activation on a soft lipid bilayer functionalized with recombinant antibody rCD2 and attached to a PDMS glass with physiological mechanical resistance (4 kPa). In this setting, while the early calcium signaling was not impacted by matrix rigidity, the late stage of activation was substrate stiffness dependent, suggesting that early steps of TCR priming do not sense the ECM mechano‐stimuli in physiological conditions.^[^ ^131^ ^]^ Nevertheless, it is well known that ECM stiffness dramatically increases in solid tumors. Therefore, Yuan et al. examined the response of human CD4+/CD8+ T cells seeded on PAA gels with a different range of stiffness (5–110 kPa) and decorated with different densities of activating antibodies (anti‐CD3, anti‐CD28) (Figure 4A). Here, it was observed that the activation and expansion are maximized at an intermediate stiffness of 25 kPa.^[^ ^133^ ^]^

Beyond activation, it was also demonstrated that ECM stiffness can also affect T‐cell proliferation, cytotoxic function, ultimately leading to exhaustion.^[^ ^132^, ^134^ ^]^ The lymph node (LN) becomes rigid and swollen during an immune response. Meng et al., shown that T cells sense these mechanical changes through the mechanosensor YAP, both in a murine LN and on a 2D alginate hydrogel scaffold with RGD peptides conjugated through carbodiimide chemistry (N‐hydroxysuccinimide (NHS)‐ 1‐Ethyl‐3‐(3‐dimethylaminopropyl) carbodiimide (EDS). Particularly, it was shown that YAP in T cells inhibits proliferation and the effector function in response to stiffness by limiting the NFAT1 translocation to the nucleus (Figure 4A).^[^ ^132^ ^]^ Only very recently, Zhang et al. demonstrated also the direct correlation between stiffness and T cell exhaustion. In their work, 2D PDMS gels with different stiffness (≈5, ≈40, and ≈200 kPa) and tethered by anti‐CD3 plus anti‐CD28 antibodies were used to demonstrate that the mechanical stress exacerbated CD8+ T cell exhaustion through thePiezo1/ Calmodulin III (CamIII)/ cAMP response element‐binding protein (CREB)/ odd‐skipped related transcription factor 2 (Osr2) signaling axis, indicating that the synergy of the costimulatory signals and matrix stiffness is critical in aggravating exhaustion (Figure 4A).^[^ ^134^ ^]^ Another platform leverages T mechanosensing, using anti‐TCR and anti‐CD28 antibody‐coated micro/nanopillars with tuneable elasticity and nano‐topography, was able to optimize and balance CAR expression, cell proliferation, central memory phenotype, CAR expression and exhaustion. This method eliminated T cell toxicity due to standard transfection and produced a class of anti‐epidermal growth factor receptor 2 (HER2) CAR able to eradicate patient‐derived xenografts (PDX) implanted subcutaneously.^[^ ^130^ ^]^

This data highlights the crucial role of biomaterials based 2D system in elucidating basic principles of T cell mechanosensing and the resulting cell response. Nevertheless, they fail to mimic the 3D environment of TME where T cells are embedded. To address this limitation, also 3D matrices have been adopted to examine the effects of mechanical properties on the T‐cell cycle.

Majedi et al. encapsulated T cells and artificial APCs (dynabeads bearing the activation signal CD3/CD28) in alginate hydrogel with stiffness 4–40 kPa. There was an augmentation of both CD4+ and CD8+ T‐cell activation, proliferation, and migration speed in the stiffer 3D hydrogel as compared to softer materials.^[^ ^123^ ^]^ In contrast, Kutzek et al. found reduced T‐cell proliferation, infiltration, and cytotoxicity, along with an increase in regulatory T‐cell markers in high‐density (4 mg mL^−1^) collagen matrix versus the low density one (1 mg mL^−1^) (Figure 4A).^[^ ^124^ ^]^ In another, Mooney's group developed a collagen hydrogel crosslinked with PEG‐norbornene, where stiffness and viscoelasticity can be tuned separately varying the collagen concentration and crosslinker amount, respectively. In this work, it was found that viscoelasticity regulates T cell and CAR‐T phenotype and function via the activator‐protein‐1 signaling pathway (AP‐1), a crucial regulator of T cell activation.^[^ ^21^ ^]^

Taken together, these data suggest that the 3D models are reliable tools to discover mechanistic insights in T cell mechanical responses.

As CD8+ and CD4+ T cells are commonly engineered for CAR‐T cell therapy, understanding the mechanical influences on these immune cells before engineering them is essential to optimize CAR‐T cell activation, proliferation, and cytotoxicity. Hence, biomaterials offer a valuable tool for investigating the mechanobiology of T cells before their use in immunotherapy. Additionally, these insights can inform priorities on mechanical features during the development of advanced preclinical models such as organoids and organ‐on‐a‐chip systems, which will be explored further below.

#### 3.3.2 Tumor Derived Organoids (TDOs) for CAR‐T Efficacy Evaluation

Once developed, CAR‐T cells cannot be effectively tested using only the 2D or even 3D models, as their efficacy depends mainly on multiple biochemical and mechanical factors rather than a single mechanical parameter. In the past decade, TDOs have emerged as a widespread 3D system for preclinical testing and drug discovery. TDOs are defined as complex, 3D ex vivo cell clusters derived from primary tumors.^[^ ^135^ ^]^ Not only do TDOs retain the genetic heterogeneity and morphological features of the tumor of origin, but can also include diverse TME cell types and ECM structure.^[^ ^136^ ^]^ Decellularized ECM, hydrogel‐based materials like, matrigels, collagen I, fibrin, and synthetic polymers like PEG, PLGA, have been implemented in organoids as they mimic the biochemical and structural support of the ECM and can be easily tunable in stiffness and composition.^[^ ^137^ ^]^Therefore, organoids offer the unprecedented opportunity to mimic the TME characteristics in vitro.

Organoids have been developed for most blood^[^ ^121^, ^138^ ^]^ and solid tumors,^[^ ^120^ ^]^ with ongoing advancements and standardization.^[^ ^139^ ^]^ Tumor‐derived organoids (TDOs) are thus ideal 3D models to assess CAR‐T efficacy against challenges in solid tumors (Table 4).

**Table 4:** Organoids and Microfluidic devices (mimicking the mechano‐TME) and used as CAR‐T screening.

System	Approach		Cancer	Source	Material	CAR‐T	Effect	Refs.
Organoids	Multi well plate		MCL	MCL biopsy	Matrigel	Anti‐CD19.CAR	Increase in tumor killing activity of CAR‐T cells	[50]
	Use of synNotch CAR‐T		Ovarian cancer	Resected OC tissues	Cold cultrex growth factor BME type 2	HER2 CAR MSLN synNotch	Increase in infiltration and anti‐tumor activity of CAR T cells	[162]
	Organoid lines of basal and luminal subtypes		Bladder cancer	Surgically resected MIBC tissues	Cold matrigel	MUC1.CAR	Specific antigen recognition, immune activation and increase in cytotoxicity	[141]
	Generated directly from fresh tumor specimens without single‐cell dissociation		Glioblastoma	Microdisected tumor	NA	2173‐BBz anti‐EGFRvIII CAR	Increase in CAR‐T cells expansion and cytotoxicity. Inability of highly specific CAR T cells to eradicate tumor cells with antigenic mutations.	[163]
	Concurrent analysis of PDOs with patients in phase I study		Glioblastoma	Surgical GBM tissue	NA	EGFR‐IL13Ra2 CAR	Cytokine release and degree of cytolysis in GBOs like patient response	[143]
System	Design	Perfusion	Cancer	Cells	Matrix	CAR‐T	Effect	
Tumor on chip	PDMS chip with tumor chambers beneath an endothealized channel.	Continuous perfusion (20 µl/h)	Breast tumor	Pleural effusion samples	PDMS, dextran‐based hydrogel	CD19.CAR	Increase in CAR‐T cell infusion, recruitment and infiltration in solid tumors	[119]
	Microfluidic device with microwells for spheroid culture connected to a fluidic channel	Static	Breast tumor	MDA‐MB‐468	3D spheroids	EGFR.CAR	Enhancement of CAR‐T cell cytotoxicity with anti‐PD‐L1 therapy and carboplatin chemotherapy	[147]
	Top fluidic chamber, porous membrane, collagen gel block and shear flow channel	Shear flow (0.25 dynes/cm^2^)	Melanoma	B16F10‐ova cells	3D collagen gel	CD8+ T cells	VEGF reduced T cell extravasation	[164]
	Bioprinter with digital micromirror device and bioink of GelMA, GMHA, photoinitiator and GBM cells	Static	Glioblastoma	SK‐N‐BE(2) neuroblastoma cells	GelMA, Glycidyl methacrylate hyaluronic acid	GD2‐CAR‐T cells	Material stiffness dampens CAR T cell infiltration and tumor cell cytotoxicity	[151]

For example, Zheng et al. used HER2+/ MSLN+ ovarian cancer organoids composed of patient tumor cells and basement membrane extract to test synNotch CAR‐T cells targeting MSLN and secreting ECM‐degrading enzymes such as metallo‐proteases (MMP)9, (MMP)‐12, and heparanase. These CAR‐T cells showed improved infiltration and antitumor activity in both organoids and a murine ovarian cancer model.^[^ ^140^ ^]^ Similarly, Yu et al. engineered CAR‐T cells against mucin 1 (MUC1), overexpressed in bladder cancer, and tested them on patient‐derived organoids using Matrigel, R‐spondin 1, and Noggin. The CAR‐T cells were selectively cytotoxic to MUC1+ organoids, demonstrating the model's utility for evaluating CAR‐T therapies in vitro.^[^ ^141^ ^]^

A key challenge of TDOs is heterogeneity from single‐cell dissociation during generation. Jacob et al. developed a glioblastoma organoid (GBO) biobank that preserved the parental tumors' histology, cellular diversity, gene expression, and mutations. In these models, anti‐ epidermal growth factor (EGFR)vIII CAR‐T cells effectively targeted tumor cells and secreted IL‐2, TNF‐α, IFN‐γ, and granzyme B.^[^ ^142^ ^]^ While TDOs are typically used preclinically, Logun et al. applied GBOs alongside a phase 1 trial of anti epidermal growth factor receptor (EGFR)IL13Rα2 CAR‐T therapy. CAR‐T cytotoxicity and cytokine release in GBOs mirrored patient cerebrospinal fluid data, supporting their utility for real‐time assessment of CAR‐T efficacy.^[^ ^143^ ^]^

Collectively, these findings affirm TDOs as a powerful platform for CAR‐T screening that accounts for tumor complexity and the TME. However, while organoids replicate ECM features, they lack other mechanical aspects like tumor microarchitecture and interstitial pressure. Innovations in microfluidics and bioprinting may help model these elements more comprehensively (Figure 1, 4).

#### 3.3.3 Microfluidic Devices and Tumor on Chips as Preclinical Models for CAR‐T Evaluation

Microfluidic technology has emerged as a superior ex vivo tumor model.^[^ ^13^ ^]^ By combining 3D gels with flow‐controlled, vascularized structures, “tumor‐on‐chip” platforms recreate cellular, biochemical, and biomechanical aspects of the TME and are widely used in drug screening, evaluating immune responses, and are widely used in drug screening. Various chip designs have been developed to study T cell migration, cytotoxicity, and immune responses.^[^ ^144^, ^145^ ^]^ In the CAR‐T context, microfluidics has primarily supported automated manufacturing steps such as cell isolation, expansion, transduction, and selection as reviewed in detail.^[^ ^146^ ^]^

Even though only a few reports are available on microfluidic models to examine cancer–immune cell interactions and assess CAR‐T efficacy, their use is in rapid expansion (Figure 4B, Table 4).

For instance, a novel breast tumor‐on‐chip model successfully replicated key aspects of CAR‐T cell function, extravasation, tumor infiltration, and cytokine release, allowing the first patient‐specific in vitro assessment of CAR‐T efficacy and safety in solid tumors like breast cancer.^[^ ^119^ ^]^The design featured a PDMS‐based chip with TDOs embedded in dextran hydrogel, loaded beneath an endothelialized channel for CAR‐T perfusion.

Microfluidic platforms using 3D spheroid co‐cultures of cancer and stromal cells have also been developed to evaluate combinatorial immunotherapies, enabling CAR‐T immunoassays. OC3D microfluidic devices (ScreenIn3D Ltd, U.K.) feature microwells connected by channels, enabling fluid flow and precise cytotoxicity and specificity testing.^[^ ^147^ ^]^

To study CAR‐T infiltration, multilayered tumor‐blood vessel chips have been created. These models feature an endothelial layer over a collagen gel with embedded tumor cells.^[^ ^148^ ^]^ Recognizing that cancer immune cell interactions are crucial for T cell recruitment, Aung et al. developed a perfusable tumor‐on‐a‐chip including cancer cells, monocytes, and endothelial cells in a gelatin hydrogel, which enhanced T cell recruitment.^[^ ^149^ ^]^

Further, the combination of microfluidics with 3D bioprinting offers high reproducibility, tunability, and scalability.^[^ ^150^ ^]^ For instance, neuroblastoma tumoroids were fabricated usingGelMA, and stiffness‐tunable glioblastoma models showed that increased ECM stiffness reduced CAR‐T penetration and cytotoxicity.^[^ ^151^ ^]^

Overall, tumor‐on‐chip platforms are powerful tools for testing CAR‐T therapies, though fully replicating the TME ex vivo remains challenging. Device customization and validation of in vivo data are essential to improve clinical relevance, with ongoing advances in tissue engineering and biomaterials driving progress.

## 4 Conclusion

With 11 products already on the market, CAR‐T represents the biggest leap forward in the field of cancer adoptive immunotherapy.^[^ ^92^ ^]^ Despite the tremendous advances in ameliorating efficacy and safety for hematological malignancies, CAR‐T‐based therapies are still dramatically limited to treat solid tumors.^[^ ^91^ ^]^ The reasons for this lack of success are multiple as solid tumors are a territory fraught with obstacles such as antigen heterogeneity and the immunosuppressive TME.^[^ ^152^ ^]^ TME not only raises a physical barrier that prevents immune cells from infiltration but also pullulates with immunosuppressive cells and soluble molecules that hijack resident T cells to a hypofunctional and exhausted state that correlates with a dismal prognosis. The obstinate efforts to create CAR‐T able to defeat the TME are interdisciplinary and involve both cell and material engineering. Synthetic biology has targeted various aspects of the TME, engineering CAR‐T cells that exploit the molecular and cellular elements to obtain an efficient anti‐tumoral response. CAR‐T armored with cytokines, with MMPs, synthetic genes circuits that sense the TME hypoxia and pH, CAR‐T directed against the vasculature, the stromal and the immune suppressive cells have proven a discrete efficacy in treating solid tumors in preclinical models.

Furthermore, synergizing CAR‐T cell engineering with biomaterials is emerging as a promising cancer treatment strategy.

Biomaterials have been used as in situ delivery systems, to bypass the obstacle of the extravasation through the tortuous and abnormal vasculature, to recreate a favorable niche for CAR‐T to proliferate and being gradually released, and as artificial APC to improve CAR‐T activation in a contest where the TAAs are difficult to reach and heterogeneous.^[^ ^77^ ^]^ The applications of fine‐tuned materials to aid the CAR‐T in navigating the hostile TME have shown increased tumor eradication in several animal models, suggesting that the cooperation of synthetic biology and biomaterial science is a promising strategy to translate CAR‐T cell immune therapy to solid tumors.

Despite these encouraging results, there are several gaps between experimental progress and their clinical implementation. Indeed, none of these studies has reached the clinical stage yet. These challenges are due to technical limitations in scale up hydrogel and particles due to batch‐to‐batch variability, high production costs, and sterility maintenance. Long term effects of CAR‐T cell‐biomaterial systems on safety and immunogenicity still remain unclear, raising the need for longer preclinical tests. Additionally, biomaterial‐based stents or needles may cause infection and trauma in the patients, therefore safer strategies are demanded. Furthermore, the lack of standardized manufactory methods and evaluation criteria hinders the regulatory and ethical approval of biomaterial‐ assisted CAR‐T delivery.^[^ ^153^ ^]^

Lastly, further molecular and biological insights on how CAR‐T cells navigate TME are needed, to shed light on their function and distribution kinetics.

A deep understanding of the biomechanical insults occurring in the TME combining genetics, epigenetics, proteomics, and metabolomics, can foster the design classes of CAR‐T that can reach this purpose, target tumor cells, and also persist the tumor‐induced premature exhaustion. This goal can be achieved using the ex vivo biomaterial‐based platforms. Indeed, 2D‐3D hydrogel systems, organoids, and tumors on chips offer the possibility to recapitulate desired characteristics of the TME alone or in concert and observe the response of T cells both from a functional and molecular point of view. These systems are already widely used to select drug combinations that overcome the TME‐induced resistance mechanisms. The immune components though, have been rarely incorporated in these systems^[^ ^154^ ^]^ while the number of studies that use cancer organoids to evaluate newly engineered CAR‐T is growing.

The contribution of the mechanical TME to CAR‐T fitness remains poorly understood. 2D and 3D hydrogels, along with microfluidic devices have been proved to be reliable tools to mimic mechanical forces and study their influence in the anti‐cancer response. These systems can integrate specific biochemical and biomechanical characteristics (solid stress, stiffened ECM, fluid pressure) and therefore represent more comprehensive preclinical models to assess the efficacy and safety of CAR‐T cells.^[^ ^155^ ^]^ The comprehensive knowledge that these systems can provide has to be further used by synthetic biology to design advanced TME‐responsive synthetic circuits that can lead to a new generation CAR‐T.

Synthetic biology, indeed, can rely on the multi‐omics studies obtained on these systems to develop sensors, synthetic receptors, or synthetic circuits that convert specific mechanic‐ TME‐clues into a favorable CAR‐T cell response that counteracts exhaustion and increases their fitness and efficacy. For instance, the knockout of the YAP and OSR2 mechano‐sensors, or a mechano‐based switch on CAR‐T, or a mechano‐sensor gene circuit that inhibits exhaustions are possible strategies to be explored. More synergistic efforts between these two fields are an open, promising possibility to be explored.

Furthermore, the use of biomaterial‐based ex vivo models at the service of CAR‐T cell engineering opens the horizon of a personalized approach where each patient's tumor is used to establish organoids that can be analyzed with multi‐omics technologies. The resulting patient specific‐tumoral and TME features can be incorporated in specific biomaterial‐based 3D systems that can be not only to test used the efficacy of existing CAR‐T but also to develop patient‐ specific CAR‐T that can navigate the prominent patient‐specific TME characteristics and successfully eradicate solid tumors.

Although all these combinatorial approaches are worthy of investigation, the high production cost remains one of the biggest challenges. Nevertheless, we envision that the synergy of interdisciplinary efforts such as those from synthetic biology and biomaterials, holds the potential of achieving the ambitious goal of developing efficient CAR‐T therapies for solid tumors.

## Conflict of Interest

The authors declare no conflict of interest.
